# The Impact of Long-Chain Omega-3 Polyunsaturated Fatty Acid Supplementation in Pregnant Women Toward the Intelligence Status of Early Childhood: Protocol for a Systematic Review and Meta-Analysis

**DOI:** 10.2196/60417

**Published:** 2025-04-17

**Authors:** Han Yin Lim, Mohammad Adi Mohammad Fadzil, Suraiami Mustar, Imanul Hassan Abdul Shukor, Wan Ahmad Syazani Mohamed

**Affiliations:** 1 Nutrition, Metabolic and Cardiovascular Research Centre Institute for Medical Research National Institutes of Health Shah Alam Malaysia; 2 Environmental Health Research Centre Institute for Medical Research National Institutes of Health Shah Alam Malaysia

**Keywords:** antenatal, long-chain omega-3 polyunsaturated fatty acids supplementation, pregnant women, systematic reviews, pregnant, pregnancy, maternal, maternity, infant, babies, nutrition, fish oil, docosahexaenoic acid, eicosapentaenoic acid, supplements, cognition, attention, motor skills, languages, behaviors, vision, neurodevelopment

## Abstract

**Background:**

Long-chain omega-3 polyunsaturated fatty acids (LCPUFAs) are essential fatty acids that protect cellular structures and provide energy, particularly for fetal growth and development. The maternal supplementations of omega-3 LCPUFA may affect the rate of intelligence in early childhood development.

**Objective:**

This systematic review aims to synthesize available evidence on the impact of omega-3 LCPUFA supplementation during pregnancy toward intelligence in early childhood development by analyzing the outcomes specifying the aspects of intelligence such as neurodevelopment, social-emotional, language, attention, behavior, cognition, vision, hearing, and motor skills.

**Methods:**

We will only include randomized controlled trials on pregnant women supplemented with omega-3 LCPUFA interventions and the outcome measured is the children’s intelligence. Based on the World Health Organization's definition of early childhood, we will include children aged 8 years or younger. Children’s intelligence can be indicated using several tools measuring their intelligence index, such as neurodevelopment, social-emotional, language, attention, behavior, cognition, vision, hearing, and motor skills. Irrelevant and unavailable studies will be excluded. A systematic search will be conducted in 3 electronic databases, namely PubMed, Scopus, and Cochrane using relevant and synonymous terms. Study screening and selection will be conducted by the authors based on eligibility criteria. Upon encountering conflicting decisions, a discussion will be held to reach a consensus. The screening and selection process will be recorded using a PRISMA (Preferred Reporting Items for Systematic Reviews and Meta-Analyses) flowchart. The included studies will be subjected to bias and quality assessment in accordance with the Critical Appraisal Skills Programme (CASP) and Grading of Recommendations Assessment, Development, and Evaluation (GRADE) assessment tool for randomized controlled trials.

**Results:**

An initial search was conducted on November 1, 2023, which returned 1998 studies for screening. The extracted data will be classified into groups and subgroups according to the indicator of intelligence measured in the study. Next, the extracted data will be summarized using tables of evidence. Whenever possible, a meta-analysis of homogeneous groups of studies will be conducted using statistical software such as RevMan (version 5.4; Cochrane Collaboration). Studies with significant heterogeneity will be discussed narratively. The systematic review is estimated to be published in November 2025.

**Conclusions:**

This systematic review will systematically pool the evidence on the potential use of omega-3 LCPUFA supplementation to improve children’s intelligence status. This review is also important in addressing any existing knowledge gaps on this topic. Finally, a deeper understanding of the association between the consumption of omega-3 LCPUFA supplementation during pregnancy and children’s intelligence will aid policy makers, health care practitioners, and mothers with more informed evidence-based decisions.

**Trial Registration:**

PROSPERO CRD42023463910; https://www.crd.york.ac.uk/PROSPERO/view/CRD42023463910

**International Registered Report Identifier (IRRID):**

DERR1-10.2196/60417

## Introduction

Long-chain omega-3 polyunsaturated fatty acids (LCPUFA), also known as omega-3 fatty acids are a part of polyunsaturated fatty acids (PUFA). It is characterized by multiple double bonds (C=C), with the first double bond located on the third carbon chain counting from the terminal methyl group (omega carbon). The 3 forms of this type of PUFA include alpha-linolenic acid (ALA), docosahexaenoic acid (DHA), and eicosapentaenoic acid (EPA). As the body lacks the necessary enzymes to synthesize ALA, it must be acquired through food or supplements, making it an essential fatty acid. Although ALA can be converted to DHA and EPA, the efficiency of its conversion is rather low, hence it is recommended to consume foods that are rich in DHA and EPA [1,2]. The primary source of ALA is plant oils such as canola oils, soybeans, and flaxseed while DHA and EPA are mainly found in fatty oil–enriched fish, which includes sardines, mackerel, and salmon [3,4].

In addition to being essential for the structural integrity of cell membranes, omega-3 LCPUFA also operates as a source of energy and bioactive lipid mediators [5,6]. These functions are critically essential for fetal growth and development, particularly for DHA as DHA plays a key role as part of the cell membrane components in the fetal brain and retina [7]. Placental transfer is the primary source of DHA accumulation in utero, which is involved in neurotransmitter metabolism, neural, and visual functions [8]. Thus, the consumption of omega-3 LCPUFA, in general, is essential to promote optimal fetal growth and maturation for several vital organs, notably the brain and eyes [9]. According to Khalid et al [10], the fetal brain and eyes that were developed in a systematic fashion in response to an adequate intake of omega-3 LCPUFA have some influence on optimal cognitive and visual acuity development in early childhood [10]. DHA prevents cognitive aging by reducing brain apoptosis through upregulating the expression of antiapoptotic proteins or downregulating the apoptotic proteins, thus enhancing early memory and learning ability related to intelligence [11].

According to the World Health Organization, the definition of early childhood corresponds to the period from prenatal development to 8 years of age [12]. While the key factors that determine intelligence in these age groups are hotly debated and vastly described, some genetic and modifiable environmental and nutritional factors contributed to the high intelligence quotient in early childhood [13,14]. Consumption of daily omega-3 LCPUFA during pregnancy as part of maternal nutritional benefits, has been linked to increased early childhood intelligence, according to multiple clinical studies [15-17]. However, as opposed to other clinical studies, randomized controlled trials (RCT) have shown that the most promising outcomes as the confounding factors will be removed in the early phase of the trial to obtain a solid causality that influences the development of early childhood intelligence.

Throughout the years, RCTs related to the consumption of omega-3 LCPUFA have reported varying degrees of results, and several systematic reviews have made an effort to assess the corpus of literature as a whole. The recent Cochrane-based systematic review of omega-3 LCPUFA conducted by Middleton et al [18] has concluded the necessity of conducting a series of follow-ups for completed RCTs for both mothers and children to understand the growth, metabolic, and neurodevelopmental pathways as the RCT outcomes varied by different types of omega-3 LCPUFA, along with the timing, doses, and characteristics of women [18]. This is also supported by a previous systematic review conducted by Gould et al [19]. However, the evidence from these reviews does not conclusively show that the consumption of omega-3 LCPUFA during pregnancy influences the improvement of cognitive and visual development in childhood. This systematic review aims to synthesize available evidence on the impact of omega-3 LCPUFA supplementation during pregnancy toward intelligence in early childhood development as compared to placebo by using a similar approach with added keywords based on the previous systematic review by Gould et al [19] and Middleton et al [18]. The outcome of children’s intelligence can be assessed using various tools that measure factors like cognition, attention, motor skills, language, behavior, vision, hearing, social-emotional, and neurodevelopment.

## Methods

### Overview

**Figure 1:**
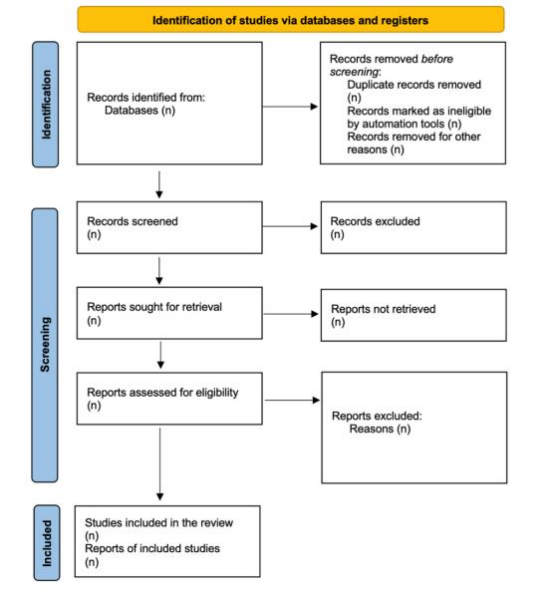
PRISMA flow diagram.

We will conduct a systematic review in accordance with the PRISMA (Preferred Reporting Items for Systematic Reviews and Meta-Analyses) [20]. We will ensure adherence to the PRISMA 2020 guidelines throughout the review process by following their checklist (Multimedia Appendix 1) and flow diagram (Figure 1). All processes will be recorded systematically, precisely, and transparently. The systematic review flow diagram will be divided into three processes: (1) identification, (2) screening, and (3) inclusion. The identification of studies will be categorized into 2 sections, via databases, registers, and other methods. Records identified from each section will be documented accordingly. The final selection process will display the total number of studies that are eligible to be included in the review. To avoid the potential confounding factors that may affect the relationship between omega-3 LCPUFA supplementation and childhood intelligence, such as socioeconomic status, maternal education, and prenatal care, several review strategies will be used in the methodology phase. First, several effect models, such as fixed effects and random effects models will be reviewed in all studies to account for unmeasured individual-level confounding that might vary over time to help in estimating the causal effects of omega-3 LCPUFA supplementation. Second, during the screening phase, only studies where the participants are randomly assigned to either the treatment group (omega-3 LCPUFA supplementation) or the placebo group will be accepted. This randomization ensures that confounders are evenly distributed between the groups, thereby reducing their potential influence on the study results.

### Eligibility Criteria

We will use the Participant, Intervention, Control, Outcomes (PICO) model in constructing eligibility criteria for study inclusion in the review.

### Study Selection

We will include individual and clustered RCTs that studied the children’s intelligence status between the supplementation of omega-3 LCPUFA during pregnancy compared to placebo or other supplemented intervention or standard perinatal care. Other types of study designs such as unpublished studies, gray literature, reviews, letters, short communications, conference papers, conference abstracts, abstracts, in vitro or in vivo, prospective or retrospective cohort, case-control, quasiexperimental and nonrandomized clinical trials will be excluded. We will include studies published in English. For studies in other languages, we will seek translations, if available.

### Population

Our systematic review will focus on studies involving healthy pregnant women who received omega-3 LCPUFA supplementation. These pregnant women must be free from major confounding factors that are not representative of the general population of pregnant women. Additionally, we will consider the outcomes for their children who are aged 8 years or younger, assessing the impact of omega-3 LCPUFA supplementation on the children’s intelligence using validated tools. The studies selected will exclusively involve mothers without chronic health conditions as well as children who have not been diagnosed with congenital anomalies, gastrointestinal issues, or metabolic disorders.

### Intervention

In the review, we will evaluate the omega-3 LCPUFA supplementation in pregnant women as the intervention. We will not put a restriction on the dose. We will follow the study authors on the dose of omega-3 LCPUFA supplementation. Any RCTs that involve the supplementation before pregnancy or during the postnatal period will be excluded.

### Comparison

We will include studies that compare the intervention with pregnant women receiving placebo or other supplement interventions.

### Outcomes

We will include studies that measured the intelligence status of children using validated inventory or tools, for example, but not limited to, The Bayley Scales of Infant and Toddler Development, Third Edition, and Kaufman Assessment Battery for Children, Second Edition. The intelligence status may be based on several indicators such as cognition, attention, motor skills, language, behavior, vision, hearing, social-emotional, and neurodevelopment. The children included in the outcome group must be aged 8 years or younger. Studies that did not measure the intelligence status of the children, or studies that used children older than 8 years as an outcome group will also be excluded.

### Data Sources, Search Terms, and Search Strategy

The authors have agreed to use 3 prominent journal databases as sources. The selected databases are PubMed, Scopus, and Cochrane Library. These databases were selected since they can fulfill every performance criterion as a search system [21]. All authors participated in a discussion to determine relevant terms and keywords that will be used in database searching. MeSH (Medical Subject Headings) terms will also be used in the search. Advanced search in each database will be performed using Boolean operators such as “AND” and “OR” to build search strings. Databases will be searched from inception to the most recent study publication. Any unpublished studies upon the final search date will not be included. No search filters will be used during the database search. The query search string will be adjusted following the interface provided by the databases (Multimedia Appendix 2).

### Data or Reference Management

All studies identified in the final hit will be downloaded using NLM or RIS format. A reference manager software, EndNote (Clarivate) will be used to combine studies identified in the 3 databases. In-apps feature using the reference manager will be used to identify and remove duplicates.

### Study Screening

Study screening will commence once database searches have been fully accomplished. A data selection form will be developed using Microsoft Excel and a systematic review tool. We will use Rayyan software (Qatar Computing Research Institute) to streamline the screening process, as it is a widely recognized tool designed for systematic reviews. All identified studies will be uploaded to the Rayyan review panel. Any duplicates identified by Rayyan will be removed. Before the process of selection, at least 5 randomly chosen studies will be pilot-tested to ensure all reviewers are familiar with the software. The references can be filtered by category, which makes the extraction process a lot easier. Collaborators or other reviewers will be invited to join the page and blind will be turned on in collaboration reviews to remove the risk of bias. References can be included by clicking “Include,” excluded by clicking “Exclude,” or “Maybe,” if not sure. The label and reason box can be used to include labels and reasons for exclusion. The probability of the references (included, excluded, or undecisive) will be calculated by Rayyan. Undecided references will have a rating from 1 to 5, where 5 is most likely to be incorporated.

The screening process will be conducted in two phases: (1) title and abstract screening, and (2) full-text screening. Two reviewers (IHAS and MAMF) will be assigned to screen the titles and abstracts based on the eligibility criteria. Following that, the other 2 reviewers (SM and WASM) will conduct the second screening process by evaluating the full-text studies to be included in the review. If both reviewers have different opinions or judgments, a third reviewer (HYL) will be called upon to unravel the disagreements.

### Data Extraction

A data extraction form will be constructed in an Excel (Microsoft Corp) sheet and the following information will be gathered from the relevant selected studies such as title, last name of the first author, year of publication, duration of intervention, study design, single or multicenter, country, source of funding, aims, population (inclusion and exclusion criteria), intervention, indicator, index test, comparison, reference standard, primary and secondary outcomes (including tools and period of measurement), and results. Two reviewers (IHAS and MAMF) will extract the data of eligible studies independently. The other 2 reviewers (SM and WASM) will recheck the data extracted. Any discrepancies will be resolved by discussion to achieve a consensus. Any missing data, incomplete information, or variation in results will be obtained by contacting the corresponding author of the study.

### Risk of Bias and Quality Assessment

Assessment of risk of bias will be conducted by 2 independent reviewers (IHAS and MAMF). A third reviewer (HYL) will be the referee for any uncertainties or disagreements in the discussion. All included RCTs will be assessed for study quality and potential biases using the Critical Appraisal Skills Programme (CASP) checklist for RCT. CASP is a checklist widely used for RCT evaluation, chosen for its simplicity, structured approach, and focus on key domains of study quality. This tool considers the following four domains of biases: (1) arising from the validity of the study design for RCT; (2) the soundness of the methodology applied; (3) measurement of the outcome; and (4) whether the results will provide any benefit to the targeted population. Each domain will be judged as “yes,” “no,” or “can’t tell.” If necessary, the authors will be contacted to obtain or clarify any information needed. The Grading of Recommendations Assessment, Development, and Evaluation (GRADE) tool will be used to assess the quality and certainty of the evidence for the outcomes analyzed in the meta-analysis. A summary of the risk of bias will be compiled into a table along with the justification for each judgement.

### Data Synthesis

Meta-analysis will be performed when there are at least 2 studies reporting on the same outcome of interest between the omega-3 supplementation (intervention) and placebo (control), otherwise, a narrative summary approach will be presented. In the case of RCT with more than 2 arms, each arm will be treated separately. Data extracted from each study will be pooled and analyzed using the RevMan (version 5.4; Cochran Collaboration) software. For outcomes of interest measured in continuous or scale, sample size, means, and corresponding SDs will be used in pooling the effect size of the outcomes across the included studies. The pooled effect estimates for continuous outcomes will be expressed as mean differences with 95% CIs for studies using a similar outcome assessment tool, meanwhile, standardized mean differences with 95% CIs will be expressed for studies using different outcome assessment tools. For outcomes of interest measured in dichotomous or binary, the number of events and total sample size will be used in pooling the effect size of the outcomes across the included studies. The pooled effect estimates for dichotomous outcomes will be expressed in odds ratio with 95% CI. In cases where the data reported in the included studies are not usable (ie, cannot be pooled with other data), the corresponding author of the study will be contacted for access to data or revised statistics. If the corresponding author is uncontactable, or the data are unavailable, we will retain the study as eligible but restricted for meta-analysis. In view of inconsistencies between results in the existing literature highlighted by Gould et al [19] and Middleton et al [18], meta-analysis will later identify patterns of agreement and disagreement, thus offering insights into the reasons behind conflicting results.

The overall effect estimates that have a *P* value less than .05 will be interpreted as statistically significant. Heterogeneity will be assessed using the *I*^2^ statistic, which represents the percentage of the total variation present between studies included. The *I*^2^ statistic values of over 50% will be considered substantial heterogeneity. To address the variability of dosage, duration, and types of PUFA supplementation, random effects models will be used during analysis to manage heterogeneity. This will ensure a robust analysis of our findings. Sensitivity analysis will be performed when heterogeneity is high to address potential confounding factors and strengthen the study’s robustness. We will group the outcomes according to the same assessment tools. Subgroup analyses for outcomes will be conducted when 2 or more studies are available per subgroup of interest. The following prespecified subgroups will be considered: maternal age, duration of intervention, presence of co-interventions (by itself or combined with complementary interventions), baseline nutritional status in mothers, sex of infant, country or geographic region, and risk of bias (low, high, or some concerns). If the dosage of the supplementation varies significantly, we will also do a subgroup analysis based on the dose group if we can pool studies with the same outcome.

Funnel plots will be conducted for the detection of publication bias if there are 10 or more studies available for an outcome. Publication bias is unlikely if data forms a symmetric inverted funnel shape around the mean effect estimate. As the funnel plots will be presented graphically, subjective judgments are required, which might differ from one person to another in interpreting the result. In addition, the Egger test will be conducted to determine funnel plot asymmetry. The presence of publication bias will be considered if the *P* value of the Egger test is less than .05.

For outcomes with insufficient data or extreme heterogeneity that cannot be analyzed by meta-analysis, a narrative synthesis will be provided.

### Registration and Reporting

This systematic review and meta-analysis protocol has been registered on the International Prospective Register of Systematic Reviews (PROSPERO, CRD42023463910) and in the National Medical Research Register Malaysia (03507-WSF). In the event of protocol amendments, the date of each amendment will be accompanied by a description of each change and the rationale on PROSPERO. In preparing this protocol, we followed the PRISMA 2020 checklist (Multimedia Appendix 1). This systematic review will be reported in accordance with the Cochrane Handbook for Systematic Reviews of Interventions [22] as well as the PRISMA-P (Preferred Reporting Items for Systematic Reviews and Meta-Analysis Protocols) guidelines (Multimedia Appendix 3) [23].

### Ethical Considerations

As this systematic review and meta-analysis uses secondary data, ethical approval is not required, and an exemption letter for ethical approval is obtained as per requirement by the Ministry of Health, Malaysia.

## Results

After discussing and finalizing the search keywords and search strategy, 1 author conducted the initial search. The first keyword search was run on November 1, 2023. The first database, which is PubMed, yielded 530 hits whereas the second database from Cochrane found 306 hits. The most hits were from Scopus with 1162 hits. In total, our first database search yielded 1998 hits. All references were then imported into a reference manager specified in the search strategy section. All hits will be pooled and screened for duplicates using automation tools. Then, the titles and abstracts will be extracted for the first screening by the authors. We aim to finish extracting and analyzing data from chosen studies and prepare the report by November 2025.

## Discussion

### Principal Findings

In our planned systematic review, we intend to investigate the existing body of literature concerning the effects of maternal omega-3 LCPUFA supplementation on the cognitive development, specifically the IQ, of children in early childhood. The justification for maternal omega-3 LCPUFA supplementation, often promoted as a means to enhance a child’s cognitive development, remains a topic of ongoing debate. Given the extensive systematic review conducted in 2018, this systematic review aims to provide a more rigorous analysis of the available evidence [18]. While previous findings suggest a positive association between omega-3 LCPUFA supplementation during pregnancy and children’s IQ, it is important to note that the existing evidence is characterized by relatively low quality due to limitations such as small sample size. Hence, our objective is to enhance the quality of evidence through this review. A consistent body of research indicates a significant link between maternal omega-3 LCPUFA supplementation during pregnancy and a substantial enhancement in the IQ of the children [24,25]. Despite the overall indication of support for the beneficial effects of maternal omega-3 LCPUFA supplementation, it is important to acknowledge the presence of factors that can influence the outcomes [26,27]. Therefore, in our systematic review, we will also look at the variations in indicators of the intelligence status of the children based on several indicators such as cognition, attention, motor, language, behavior, vision, hearing, social-emotional, and neurodevelopment, which may contribute to heterogeneity in the results. The systematic review will adhere strictly to the protocol, and any deviations will be documented and reported in the published manuscript.

The implications of these findings for public health are significant. A more tailored approach to prenatal nutritional guidance, as suggested by Cetin et al [28], may be warranted to ensure optimal cognitive development in early childhood [28]. This, in turn, could have long-term cost-saving benefits for health care systems by potentially reducing the societal burden of cognitive impairments.

### Comparison With Prior Work

Previous systematic reviews were published more than 5 years ago and the findings were varied [18,19]. Given the potential impact of maternal omega-3 LCPUFA supplementation on child IQ, there is a strong case for revisiting and possibly refining dietary recommendations for pregnant women. Thus, this review builds on these works by incorporating updated evidence and conducting subgroup analyses to address key factors, such as timing, dosage, and source of supplementation.

### Limitations

We will thoroughly examine the limitations and potential sources of bias in the included studies. Despite our review’s rigorous evaluation of evidence quality, there may still be sources of bias, such as publication bias or unaccounted confounders, that influence the overall results. To address potential publication bias, we will include a funnel plot analysis to visually assess the presence of asymmetry. Furthermore, the quality and design of the individual studies may vary. It is important for researchers and policymakers to recognize these limitations when interpreting the findings and consider them when making recommendations.

### Future Research

This systematic review will address the necessity for further research in this area. It will also help future studies to address the identified limitations and clarify the optimal timing, dosage, and duration of maternal omega-3 LCPUFA supplementation to maximize the potential benefits on child IQ. Additionally, the long-term effects of maternal supplementation in children beyond the age of 8 years warrant exploration. This will not only deepen our understanding of this complex relationship but also help refine practical recommendations for expectant mothers, contributing to the well-being of future generations.

### Conclusions

In conclusion, the systematic review will provide valuable insights into the potential impact of maternal omega-3 LCPUFA supplementation on children’s IQ. If the evidence suggests a positive association, further research is needed to address limitations, confirm findings, and refine recommendations. The results will be disseminated by publishing the review, presenting at conferences, and sharing with stakeholders such as policy makers, nutritionists, and doctors, following PRISMA guidelines for transparent reporting. The implications for public health are substantial, and addressing these gaps can lead to a more comprehensive exploration of the impact of omega-3 LCPUFA supplementation during pregnancy on children’s intelligence. Thus, this systematic review can provide a thorough understanding of the topic and offer valuable insights for future research and practice.

## Appendix

Multimedia Appendix 1 PRISMA 2020 checklist.

Multimedia Appendix 2 The protocol search strategy.

Multimedia Appendix 3 PRISMA-P 2015 checklist.

## Abbreviations

ALA: alpha-linolenic acid
CASP: Critical Appraisal Skills Programme
DHA: docosahexaenoic acid
EPA: eicosapentaenoic acid
GRADE: Grading of Recommendations Assessment, Development, and Evaluation
LCPUFA: long-chain omega-3 polyunsaturated fatty acid
MeSH: Medical Subject Headings
PICO: Participant, Intervention, Control, Outcomes
PRISMA: Preferred Reporting Items for Systematic Reviews and Meta-Analysis
PRISMA-P: Preferred Reporting Items for Systematic Reviews and Meta-Analysis Protocols
PUFA: polyunsaturated fatty acid
RCT: randomized controlled trial

## Data Availability

All data generated and analyzed in this systematic review are available upon request from the corresponding author.
