# Supplementary material for: The Impact of Long-Chain Omega-3 Polyunsaturated Fatty Acid Supplementation in Pregnant Women Toward the Intelligence Status of Early Childhood: Protocol for a Systematic Review and Meta-Analysis
Source: JMIR Res Protoc. 2025 Apr 17;14:e60417. doi: 10.2196/60417 (PMC12046255; doi:10.2196/60417)

**Multimedia Appendix 1**

**Preferred Reporting Items for Systematic Reviews and Meta-Analyses (PRISMA) 2020 Checklist.**

**_________________________________________________________________________________**
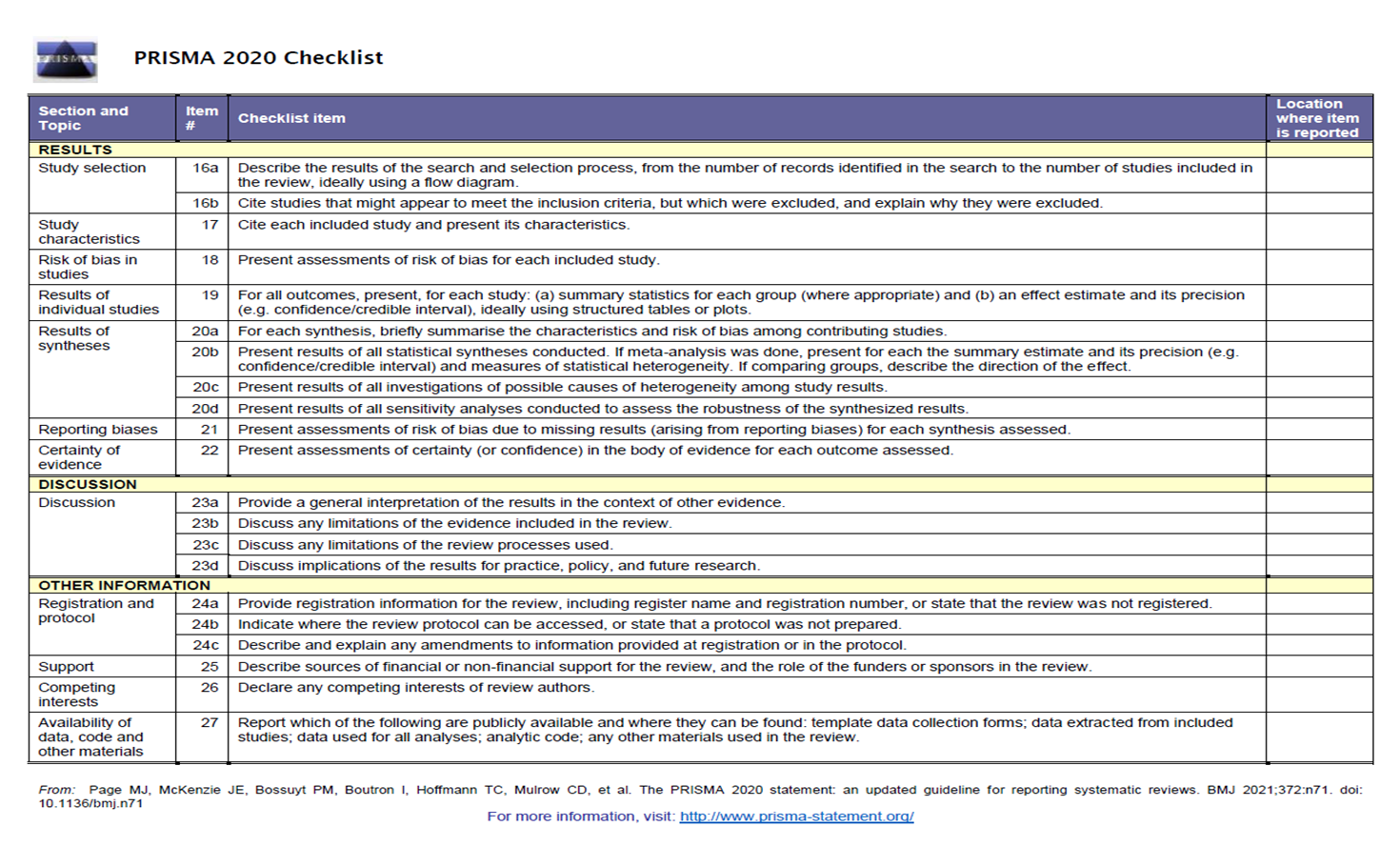

Supplement: Multimedia Appendix 1 [file resprot_v14i1e60417_app1.docx]
