# Supplementary material for: The Impact of Long-Chain Omega-3 Polyunsaturated Fatty Acid Supplementation in Pregnant Women Toward the Intelligence Status of Early Childhood: Protocol for a Systematic Review and Meta-Analysis
Source: JMIR Res Protoc. 2025 Apr 17;14:e60417. doi: 10.2196/60417 (PMC12046255; doi:10.2196/60417)
Supplement: Multimedia Appendix 2 [file resprot_v14i1e60417_app2.docx]

**Multimedia Appendix 2: Search Strategy**

**Pubmed**

| Time: 6/11/2023 10:20:58 AM | |  |
| --- | --- | --- |
| Search number | Query | Results |
| 5 | (((((((((((((Pregnancy[MeSH Terms]) OR (Maternal[Title/Abstract])) OR (Prenatal[Title/Abstract])) OR (Pregnant[Title/Abstract])) OR (Transplacental[Title/Abstract])) OR (Placenta[Title/Abstract])) OR (Pregnancy[Title/Abstract])) OR (Gestation[Title/Abstract])) OR (Antenatal[Title/Abstract])) OR (Fetal[Title/Abstract])) OR (Fetus[Title/Abstract])) AND (((((((((clinical trials, randomized[MeSH Terms]) OR (Randomized[Title/Abstract])) OR (Trial[Title/Abstract])) OR (Clinical[Title/Abstract])) OR (Supplement[Title/Abstract])) OR (Treatment[Title/Abstract])) OR (Intervention[Title/Abstract])) OR (Placebo[Title/Abstract])) OR (Control[Title/Abstract]))) AND (((((((((((acids, polyunsaturated fatty[MeSH Terms]) OR (Docosahexaenoic acid[Title/Abstract])) OR (Docosahexaenoate[Title/Abstract])) OR (omega 3[Title/Abstract])) OR (n-3[Title/Abstract])) OR (LCPUFA[Title/Abstract])) OR (PUFA[Title/Abstract])) OR (long chain polyunsaturated fatty acid[Title/Abstract])) OR (fish oil[Title/Abstract])) OR (marine oil[Title/Abstract])) OR (algal oil[Title/Abstract]))) AND ((((((((((((((((((((Intelligence[MeSH Terms]) OR (Cognitive[Title/Abstract])) OR (Cognition[Title/Abstract])) OR (Neurologic[Title/Abstract])) OR (Neurodevelopment[Title/Abstract])) OR (Psychological[Title/Abstract])) OR (Phychology[Title/Abstract])) OR (Intelligence[Title/Abstract])) OR (IQ[Title/Abstract])) OR (Intelligent[Title/Abstract])) OR (Visual Acuity[Title/Abstract])) OR (Visual evoked potential[Title/Abstract])) OR (VEP[Title/Abstract])) OR (Visual development[Title/Abstract])) OR (Retina[Title/Abstract])) OR (Retinal[Title/Abstract])) OR (Teller[Title/Abstract])) OR (Behavioral acuity[Title/Abstract])) OR (Stereoacuity[Title/Abstract])) OR (Electroretinogram[Title/Abstract])) | 530 |
| 4 | (((((((((((((((((((Intelligence[MeSH Terms]) OR (Cognitive[Title/Abstract])) OR (Cognition[Title/Abstract])) OR (Neurologic[Title/Abstract])) OR (Neurodevelopment[Title/Abstract])) OR (Psychological[Title/Abstract])) OR (Phychology[Title/Abstract])) OR (Intelligence[Title/Abstract])) OR (IQ[Title/Abstract])) OR (Intelligent[Title/Abstract])) OR (Visual Acuity[Title/Abstract])) OR (Visual evoked potential[Title/Abstract])) OR (VEP[Title/Abstract])) OR (Visual development[Title/Abstract])) OR (Retina[Title/Abstract])) OR (Retinal[Title/Abstract])) OR (Teller[Title/Abstract])) OR (Behavioral acuity[Title/Abstract])) OR (Stereoacuity[Title/Abstract])) OR (Electroretinogram[Title/Abstract]) | 1,378,655 |
| 3 | ((((((((((acids, polyunsaturated fatty[MeSH Terms]) OR (Docosahexaenoic acid[Title/Abstract])) OR (Docosahexaenoate[Title/Abstract])) OR (omega 3[Title/Abstract])) OR (n-3[Title/Abstract])) OR (LCPUFA[Title/Abstract])) OR (PUFA[Title/Abstract])) OR (long chain polyunsaturated fatty acid[Title/Abstract])) OR (fish oil[Title/Abstract])) OR (marine oil[Title/Abstract])) OR (algal oil[Title/Abstract]) | 354,906 |
| 2 | ((((((((clinical trials, randomized[MeSH Terms]) OR (Randomized[Title/Abstract])) OR (Trial[Title/Abstract])) OR (Clinical[Title/Abstract])) OR (Supplement[Title/Abstract])) OR (Treatment[Title/Abstract])) OR (Intervention[Title/Abstract])) OR (Placebo[Title/Abstract])) OR (Control[Title/Abstract]) | 11,192,196 |
| 1 | ((((((((((Pregnancy[MeSH Terms]) OR (Maternal[Title/Abstract])) OR (Prenatal[Title/Abstract])) OR (Pregnant[Title/Abstract])) OR (Transplacental[Title/Abstract])) OR (Placenta[Title/Abstract])) OR (Pregnancy[Title/Abstract])) OR (Gestation[Title/Abstract])) OR (Antenatal[Title/Abstract])) OR (Fetal[Title/Abstract])) OR (Fetus[Title/Abstract]) | 1,401,744 |

**Scopus:**

| Date: 6-Nov-2023 | | |
| --- | --- | --- |
| Search number | Query | Results |
| 5 | ((TITLE-ABS-KEY(pregnant) OR TITLE-ABS-KEY(maternal) OR TITLE-ABS-KEY(prenatal) OR TITLE-ABS-KEY(transplacental) OR TITLE-ABS-KEY(placenta) OR TITLE-ABS-KEY(pregnancy) OR TITLE-ABS-KEY(gestation) OR TITLE-ABS-KEY(antenatal) OR TITLE-ABS-KEY(fetal) OR TITLE-ABS-KEY(fetus))) AND ((TITLE-ABS-KEY(randomized) OR TITLE-ABS-KEY(trial) OR TITLE-ABS-KEY(clinical) OR TITLE-ABS-KEY(supplement) OR TITLE-ABS-KEY(treatment) OR TITLE-ABS-KEY(intervention) OR TITLE-ABS-KEY(placebo) OR TITLE-ABS-KEY(control))) AND ((TITLE-ABS-KEY(docosahexaenoic) OR TITLE-ABS-KEY(docosahexaenoate) OR TITLE-ABS-KEY(omega 3) OR TITLE-ABS-KEY(n-3) OR TITLE-ABS-KEY(lcpufa) OR TITLE-ABS-KEY(pufa) OR TITLE-ABS-KEY(long AND chain AND polyunsaturated AND fatty AND acid) OR TITLE-ABS-KEY(fish AND oil) OR TITLE-ABS-KEY(marine AND oil) OR TITLE-ABS-KEY(algal AND oil))) AND ((TITLE-ABS-KEY(cognitive) OR TITLE-ABS-KEY(cognition) OR TITLE-ABS-KEY(neurologic) OR TITLE-ABS-KEY(neurodevelopment) OR TITLE-ABS-KEY(psychological) OR TITLE-ABS-KEY(psychology) OR TITLE-ABS-KEY(intelligence) OR TITLE-ABS-KEY(iq) OR TITLE-ABS-KEY(intelligent) OR TITLE-ABS-KEY(visual AND acuity) OR TITLE-ABS-KEY(visual AND evoked AND potential) OR TITLE-ABS-KEY(vep) OR TITLE-ABS-KEY(visual AND development) OR TITLE-ABS-KEY(retina) OR TITLE-ABS-KEY(retinal) OR TITLE-ABS-KEY(teller) OR TITLE-ABS-KEY(behavioral AND acuity) OR TITLE-ABS-KEY("stereoacuity") OR TITLE-ABS-KEY(electroretinogram))) | 1162 |
| 4 | (TITLE-ABS-KEY(cognitive) OR TITLE-ABS-KEY(cognition) OR TITLE-ABS-KEY(neurologic) OR TITLE-ABS-KEY(neurodevelopment) OR TITLE-ABS-KEY(psychological) OR TITLE-ABS-KEY(psychology) OR TITLE-ABS-KEY(intelligence) OR TITLE-ABS-KEY(iq) OR TITLE-ABS-KEY(intelligent) OR TITLE-ABS-KEY(visual AND acuity) OR TITLE-ABS-KEY(visual AND evoked AND potential) OR TITLE-ABS-KEY(vep) OR TITLE-ABS-KEY(visual AND development) OR TITLE-ABS-KEY(retina) OR TITLE-ABS-KEY(retinal) OR TITLE-ABS-KEY(teller) OR TITLE-ABS-KEY(behavioral AND acuity) OR TITLE-ABS-KEY("stereoacuity") OR TITLE-ABS-KEY(electroretinogram)) | 5,116,955 |
| 3 | (TITLE-ABS-KEY(docosahexaenoic) OR TITLE-ABS-KEY(docosahexaenoate) OR TITLE-ABS-KEY(omega 3) OR TITLE-ABS-KEY(n-3) OR TITLE-ABS-KEY(lcpufa) OR TITLE-ABS-KEY(pufa) OR TITLE-ABS-KEY(long AND chain AND polyunsaturated AND fatty AND acid) OR TITLE-ABS-KEY(fish AND oil) OR TITLE-ABS-KEY(marine AND oil) OR TITLE-ABS-KEY(algal AND oil)) | 373,398 |
| 2 | (TITLE-ABS-KEY(randomized) OR TITLE-ABS-KEY(trial) OR TITLE-ABS-KEY(clinical) OR TITLE-ABS-KEY(supplement) OR TITLE-ABS-KEY(treatment) OR TITLE-ABS-KEY(intervention) OR TITLE-ABS-KEY(placebo) OR TITLE-ABS-KEY(control)) | 25,604,586 |
| 1 | (TITLE-ABS-KEY(pregnant) OR TITLE-ABS-KEY(maternal) OR TITLE-ABS-KEY(prenatal) OR TITLE-ABS-KEY(transplacental) OR TITLE-ABS-KEY(placenta) OR TITLE-ABS-KEY(pregnancy) OR TITLE-ABS-KEY(gestation) OR TITLE-ABS-KEY(antenatal) OR TITLE-ABS-KEY(fetal) OR TITLE-ABS-KEY(fetus)) | 1,828,081 |

**Cochrane:**

| Date Run: 6/11/2023 4:59:45 PM  Comment: | | |
| --- | --- | --- |
| ID | Search | Hits |
| #1 | MeSH descriptor: [Pregnancy] explode all trees | 31569 |
| #2 | (Pregnant):ti,ab,kw OR (Maternal):ti,ab,kw OR (Prenatal):ti,ab,kw OR (Transplacental):ti,ab,kw OR (Placenta):ti,ab,kw | 51,527 |
| #3 | (Pregnancy):ti,ab,kw OR (Gestation):ti,ab,kw OR (Antenatal):ti,ab,kw OR (Fetal):ti,ab,kw OR (Fetus):ti,ab,kw | 83,475 |
| #4 | #1 OR #2 OR #3 | 100,077 |
| #5 | MeSH descriptor: [Randomized Controlled Trial] explode all trees | 25,732 |
| #6 | (Randomized):ti,ab,kw OR (Trial):ti,ab,kw OR (Clinical):ti,ab,kw OR (Supplement):ti,ab,kw OR (Treatment):ti,ab,kw | 1627258 |
| #7 | (Intervention):ti,ab,kw OR (Placebo):ti,ab,kw OR (Control):ti,ab,kw | 1093774 |
| #8 | #5 OR #6 OR #7 | 1794469 |
| #9 | MeSH descriptor: [Fatty Acids, Unsaturated] explode all trees | 14437 |
| #10 | (Docosahexaenoic acid):ti,ab,kw OR (Docosahexaenoate):ti,ab,kw OR (Omega 3):ti,ab,kw OR (n-3):ti,ab,kw OR (LCPUFA):ti,ab,kw | 15600 |
| #11 | (PUFA):ti,ab,kw OR (Long chain polyunsaturated fatty acid):ti,ab,kw OR (Fish oil):ti,ab,kw OR (Marine oil):ti,ab,kw OR (Algal oil):ti,ab,kw | 5767 |
| #12 | #9 OR #10 OR #11 | 27762 |
| #13 | MeSH descriptor: [Intelligence] explode all trees | 8284 |
| #14 | (Cognitive):ti,ab,kw OR (Cognition):ti,ab,kw OR (Neurologic):ti,ab,kw OR (Neurodevelopment):ti,ab,kw OR (Psychological):ti,ab,kw | 160742 |
| #15 | (Psychology):ti,ab,kw OR (Intelligence):ti,ab,kw OR (IQ):ti,ab,kw OR (Intelligent):ti,ab,kw OR (Visual acuity):ti,ab,kw | 107017 |
| #16 | (Visual evoked potential):ti,ab,kw OR (VEP):ti,ab,kw OR (Visual development):ti,ab,kw OR (Retina):ti,ab,kw OR (Retinal):ti,ab,kw | 16400 |
| #17 | (Teller):ti,ab,kw OR (Behavioral acuity):ti,ab,kw OR (Stereoacuity):ti,ab,kw OR (Electroretinogram):ti,ab,kw | 797 |
| #18 | #13 OR #14 OR #15 OR #16 OR #17 | 240829 |
| #19 | #4 AND #8 AND #12 AND #18 | 306 |
